# Genotypes and Phenotypes: A Search for Influential Genes in Diabetic Retinopathy

**DOI:** 10.3390/ijms21082712

**Published:** 2020-04-14

**Authors:** Andrea P. Cabrera, Rushi N. Mankad, Lauren Marek, Ryan Das, Sampath Rangasamy, Finny Monickaraj, Arup Das

**Affiliations:** 1 Department of Surgery, University of New Mexico School of Medicine, Albuquerque, NM 87131, USA; apcabrera@salud.unm.edu (A.P.C.); RMankad@salud.unm.edu (R.N.M.); lmarek@salud.unm.edu (L.M.); RyanDas5@gmail.com (R.D.); fmonicaraj@salud.unm.edu (F.M.); 2 Translational & Genomics Research Institute, Phoenix, AZ 85004, USA; srangasamy@tgen.org; 3 New Mexico VA Health Care System, Albuquerque, NM 87108, USA

**Keywords:** genomics, genotype, phenotype, diabetic retinopathy, diabetes

## Abstract

Although gene–environment interactions are known to play an important role in the inheritance of complex traits, it is still unknown how a genotype and the environmental factors result in an observable phenotype. Understanding this complex interaction in the pathogenesis of diabetic retinopathy (DR) remains a big challenge as DR appears to be a disease with heterogenous phenotypes with multifactorial influence. In this review, we examine the natural history and risk factors related to DR, emphasizing distinct clinical phenotypes and their natural course in retinopathy. Although there is strong evidence that duration of diabetes and metabolic factors play a key role in the pathogenesis of DR, accumulating new clinical studies reveal that this disease can develop independently of duration of diabetes and metabolic dysfunction. More recently, studies have emphasized the role of genetic factors in DR. However, linkage analyses, candidate gene studies, and genome-wide association studies (GWAS) have not produced any statistically significant results. Our recently initiated genomics study, the Diabetic Retinopathy Genomics (DRGen) Study, aims to examine the contribution of rare and common variants in the development DR, and how they can contribute to clinical phenotype, rate of progression, and response to available therapies. Our preliminary findings reveal a novel set of genetic variants associated with proangiogenic and inflammatory pathways that may contribute to DR pathogenesis. Further investigation of these variants is necessary and may lead to development of novel biomarkers and new therapeutic targets in DR.

## 1. Introduction

In 1996, a group of researchers reported that a cohort of 25 individuals remained uninfected with human immunodeficiency virus type 1 (HIV-1) in spite of multiple high-risk sexual exposures [1]. How did they get “protected” in spite of the environmental risk factors? This resistance to the virus was attributed to a protective mutation in the C-C Motif Chemokine Receptor 5 (*CCR5*) gene, a co-receptor for HIV-1 [2]. People who carry this gene mutation are thereby protected from the deadly infection because the mutation results in a 32 base-pair deletion rendering *CCR5* undetectable on the cell surface and is therefore impenetrable by the virus in homozygous individuals. Based on this mechanism, a variety of novel anti-HIV strategies ranging from oral *CCR5* antagonists to the controversial RNA-guided clustered regularly interspaced short palindromic repeats genome editing (CRISPR/Cas9) of *CCR5* have been explored [3]. This kind of genetic mutation that protects one against disease raises the fundamental question about how *genotype*, the genetic makeup of an organism, determines its *phenotype*, the observable characteristics, that are to some extent, controlled by environmental variables. It is in this way that identical genotypes can express different phenotypes as the result of unique interactions with the environment, much like we see in cases where both twins do not always manifest genetically determinable disease.

We have come a long way from the days of Gregor Mendel, a monk who did the most important biology experiments in a monastery garden of pea plants and discovered the laws of heredity. Since then, we have gone from the discovery of genes, to the discovery of DNA by Watson and Crick, to the completion of the Human Genome Project. However, it was Theodosius Dobzhansky, a Ukrainian biologist at Columbia University, who first noted that genotypes were not the sole determinants of phenotypes, but the environment also contributed to the organism’s physical attributes [4,5]. This fundamental concept highlights important equation in biology: *Genotype* + Environment = *Phenotype*. Today, it is widely accepted that gene–environment interactions play a fundamental role in the inheritance of complex traits in both health and disease. However, precisely how a *genotype* and the environmental cues lead to an observable *phenotype,* remains to be elucidated. Many questions remain: In the presence of a gene mutation, what is the likelihood that disease will develop? Is the environment sufficient to cause phenotypic changes? Are “external triggers” on the genotype necessary for phenotype changes to manifest? Thus, this equation can be further modified into: *Genotype* + Environment + “External Triggers” = *Phenotype*. The breast cancer type 1 gene (*BRCA1*), first discovered in 1994, demonstrated this phenomenon that genotypes require environmental cues and external triggers to manifest into an observable phenotype [6,7]. In this case, a mutant *BRCA1* gene is not sufficient to increase the risk of breast cancer and thus not all women carrying the *BRCA1* mutation develop cancer [8]. These genes, having incomplete “penetrance,” only become expressed when there are “external triggers” present.

## 2. Role of Genetic Variance in Complex Diseases

In the last decade, the field of genetics has provided new frontiers in investigating mechanisms of human disease. The identification of novel genetic variants that influence pathogenesis not only yield promise of efficacious therapeutic targets but also give rise to a path toward disease prevention. The ophthalmology field in particular has already witnessed milestones leading to direct clinical translation of genomic discoveries. For instance, genetic variant information has been used to identify genotype-phenotype links such as the *HF1/CFH* polymorphism conferring 50% risk in age-related macular degeneration [9,10,11,12]. Moreover, *restoration* of vision is now achievable via targeted gene therapy as in cases of Biallelic RPE65 mutation-associated retinal dystrophy [13]. More recently, gene mapping has been utilized to identify novel genetic variants underlying diabetic retinopathy (DR) [14,15,16,17,18,19,20]. However, only weak associations have resulted. Although DR-associated genes have yet to be confirmed, these early findings represent the initial groundwork and may be a preview of the complexity underlying DR genetics.

Understanding the fundamental role of genetic variance in complex diseases has been historically difficult [21,22]. In the case of DR, the complexity of this disease presents a unique challenge because it manifests with heterogenous phenotypes, has a myriad of biochemical pathways associated, and is a complication of another complex disease (diabetes). However, these challenges are not unique to retinopathy. Importantly, the successful identification of genetic associations in other similarly complex diseases have been centered around phenotypic variation [23,24,25]. By carefully identifying and leveraging heterogenous phenotypes found in a particular disease, key variants conferring risk have been identified and have furthered our understanding of disease pathogenesis.

However, relatively little work has been carried out to delineate phenotypes in the study of DR genetics. We speculate lack of emphasis on the phenotypic variation as the major obstacle in elucidating the role of genetic variance in this disease. Thus, in this review, we examine the natural history and risk factors associated with DR, emphasizing rare and discrete clinical phenotypes that manifest in retinopathy. Additionally, we propose how a careful delineation of clinical phenotypes in DR can be leveraged to answer questions that have yet to be addressed but cannot be explained by our current understanding of the risk factors associated. Lastly, we introduce our genomics initiative: Diabetic Retinopathy Genetics (DRGen) Study, a multi-center collaborative effort aimed at the identification of rare and common genetic variants associated with the distinct clinical phenotypes and pharmacological responses observed in DR patients.

## 3. Natural History and Clinical Phenotypes of Diabetic Retinopathy

Diabetic retinopathy (DR) is the most common microvascular complication of diabetes. At present, nearly 100% of patients with type 1 diabetes and approximately 60% of patients with type 2 diabetes will develop some form of retinopathy within the first two decades of diabetes onset [26]. Large-scale epidemiological studies show that duration of diabetes and total glycemic exposure are the *strongest* risk factors for DR [27,28]. As a result, there is tremendous emphasis centered around intensive glucose management. However, while the role of glucose control has been demonstrated in slowing disease progression, accumulating clinical studies reveal DR can develop independently of metabolic dysfunction [29,30,31]. Additionally, emerging data on continuous glucose monitoring systems, indicate that risk factors *not including* average glucose value (HbA1c) are involved in chronic macro- and micro- complications of diabetes, challenging the canonical role of glucose as a major cause to the onset and progression of retinopathy [32]. Currently, the precise mechanisms underlying pathogenesis are not well understood. The precedence, complexity, and prognosis of debilitating vision loss emphasize the need to search for other factors contributing to DR.

When the advent of the ophthalmoscope first enabled visualization of macular anomalies, the causal relationship between retinal complications and diabetes was speculative [33]. It would take 14 years from the first observation until histopathological proof would emerge, ultimately solidifying the causal relationship between retinal complications and diabetes [34]. Later, the discovery of insulin would transform diabetes care, providing the first effective treatment for diabetes management and consequently increasing the prevalence and phenotypic diversity of DR (and other complications) [35]. Recognizing the need to classify the diverse phenotypes, the Airlie House Symposium (1968) gave rise to a sophisticated system, which is still used today. Based on fundus photographs, a modified Airlie House classification, distinguishes clinical features on a scale ranging from no disease to severe retinopathy [36]. Generally, DR is considered to progress from no disease, to nonproliferative diabetic retinopathy (NPDR), culminating in the more advanced proliferative diabetic retinopathy (PDR) [37,38]. This classification system additionally includes distinguishing features of diabetic macular edema (DME) including a subset of mild, moderate, and severe cases that can occur concurrently or independent of either NPDR or PDR. Once a diagnosis is established, disease progression rates vary widely between individuals [39,40,41].

### 3.1. Nonproliferative Diabetic Retinopathy (NPDR)

**Figure 1:**
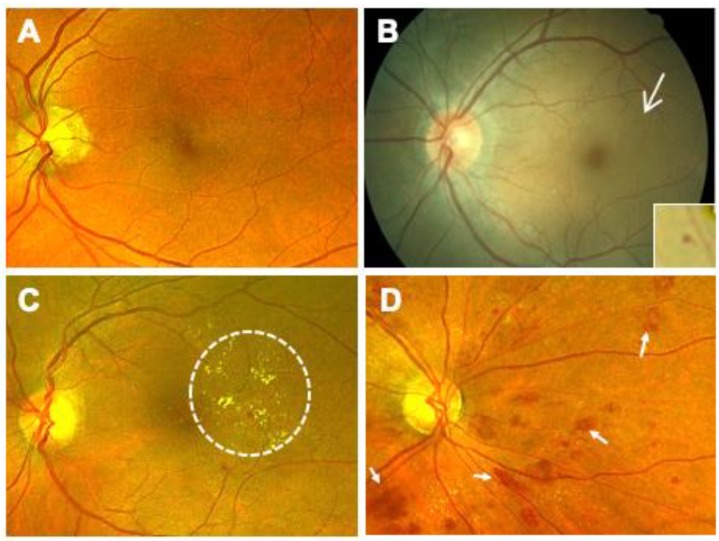
Stages of Progressing Nonproliferative Diabetic Retinopathy (NPDR). (**A**) Fundus photograph of a retina with no retinopathy observed during the first 10 years of diabetes. (**B**) In Mild NPDR, the first detectible sign of disease consists of a single microaneurysm (arrow, inset). (**C**) In moderate NPDR, retinal hemorrhages and hard exudates (circled) may be observed. (**D**) In severe NPDR, >20 intraretinal hemorrhages may be observed throughout the retina. Venous beading and intraretinal microvascular anomalies (IRMA) may be also seen (arrows).

Mild nonproliferative diabetic retinopathy (NPDR) is the earliest stage of diabetic retinal disease (Figure 1A). After a variable period of no retinopathy typically lasting 8–10 years, the first detectible sign of disease consists of a single microaneurysm with no other visible lesions or abnormalities (Figure 1B). The selective loss of retinal pericytes in the retinal capillaries, a hallmark of early DR, results in focal outpouching and possible endothelial cell proliferation [42]. Mild NPDR is left untreated, with careful annual follow-up examination to monitor disease progression. As the disease progresses into moderate NPDR, additional microaneurysms will appear along with other vascular changes like intraretinal hemorrhages and hard exudates (Figure 1C). During the more severe NPDR, more than 20 intraretinal hemorrhages can be observed throughout the entirety of the retina along with other features like venous beading and intraretinal microvascular anomalies (IRMA) (Figure 1D). Patients with severe NPDR are at a 52% increased risk to advance to proliferative diabetic retinopathy (PDR) within one year [43].

### 3.2. Proliferative Diabetic Retinopathy (PDR)

**Figure 2:**
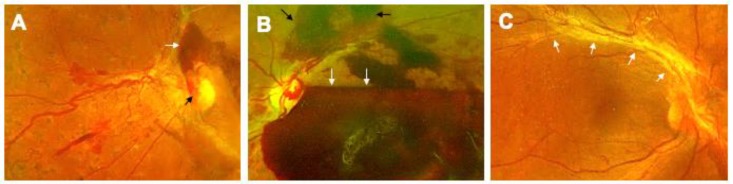
Progressive Stages of Proliferative Diabetic Retinopathy (PDR). (**A**) The proliferative stage of diabetic retinopathy is marked by neovascularization, visible on the optic nervehead (black arrow). Additionally, preretinal hemorrhage on the superior aspect of the nerve can also be seen (white arrow). (**B**) A subhyaloid hemorrhage (white arrows) manifests with boat-shaped configuration as it is trapped in the potential space between the posterior hyaloid and the internal limiting membrane. Preretinal hemorrhages can also be observed (black arrows). (**C**) Another complication occurring in this stage is the formation of a fibrovascular band along the superotemporal arcade, which can contract, causing tractional retinal detachment (white arrows).

The proliferation of new vessels, termed neovascularization, is the major structural change evident in proliferative diabetic retinopathy (PDR; Figure 2A). Newly formed vessels growing on the surface of the retina, can easily bleed into the vitreous cavity, causing preretinal and vitreous hemorrhage and further heightening the risk for vision loss (Figure 2B) [44,45]. In more severe cases, the fibro-vascular tissue can grow along with vessels, and contract causing traction retinal detachment (TRD; Figure 2C). Vision loss in PDR patients can be halted by one of the several available treatment options (anti-vascular endothelial growth factor (VEGF) injections, panretinal photocoagulation laser, or pars plana vitrectomy) [46,47,48,49]. PDR occurs in about 50% of patients with type 1 diabetes, and about 10% patients with type 2 diabetes after 15 years of duration of diabetes [26,27]. It has been observed that even in spite of longer duration of diabetes (up to 50 years), the prevalence of PDR in diabetics remains at 50% in type 1 diabetics. Why the other 50% patients do not develop PDR in their lifetime remains unknown.

### 3.3. Diabetic Macular Edema (DME)

**Figure 3:**
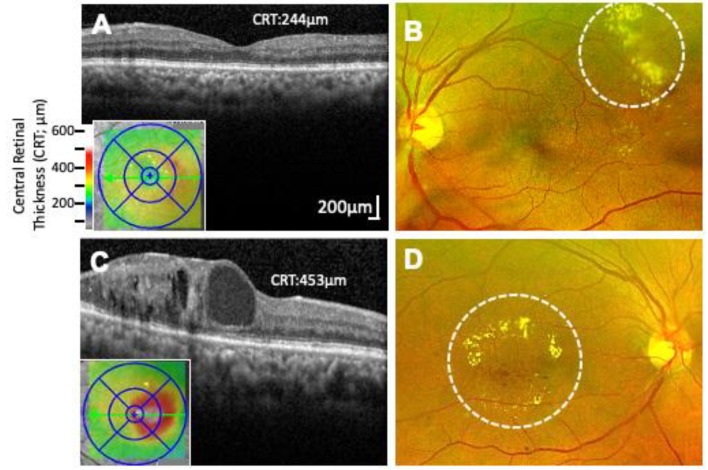
Non-center and Center Involving Macular Edema. (**A**) Optical coherence tomography (OCT) image of the macular region of the retina showing non-center involving macular edema (Central retinal thickness, 244 µm). (**B**) Corresponding fundus photograph of (A) showing characteristic exudates formed outside of the macular region (marked by dashed circle). (**C**) OCT image of center involving macular edema with cystic changes within the retina (Central retinal thickness, 453 µm). (**D**) Corresponding fundus photograph of C showing characteristic hard exudates and edema formed inside the macula (marked by dashed circle).

Diabetic macular edema (DME) is the leading cause of vision loss among diabetics [50,51,52]. Characterized by the breakdown of the blood–retinal barrier (BRB) resulting from pericyte loss, basement membrane thickening, and breakdown of endothelial cell junctions, DME can develop independently at any stage of DR (nonproliferative and proliferative) [53,54]. The severity (mild, moderate, or severe) is based on a combination of observable factors. In the mild and moderate stages, hard exudates (deposition of lipids from plasma), and capillary lesions resulting in leakage from plasma, form distant from the macula (non-center-involving DME), as confirmed by optical coherence tomography (OCT; Figure 3A,B) [55]. As severity progresses through the moderate stage, increased retinal thickening and hard exudates begin to encroach the macula (center-involving DME), causing vision loss (Figure 3C,D). Severe DME results when capillary leakage and increasing number of center-involving hard exudates lead to extensive destruction of the BRB [55]. In patients with non-center involving DME, because there is no vision loss, the treatment is often observation though focal/grid laser photocoagulation may be offered. In patients with center-involving DME, the treatment option depends on the visual acuity as currently recommended by the Diabetic Retinopathy Clinical Research Network (DRCR.net) protocol V [56]. With visual acuity of 20/20–20/25, the recommendation is observation, whereas if it reaches or exceeds 20/30, the first line of treatment is intravitreal anti-vascular endothelial growth factor (anti-VEGF) injections. The incidence of DME after 10 years of diabetes is 20.1% in type 1 diabetics, 25.4% in type 2 diabetics requiring insulin, and 13.9% in type 2 diabetics not requiring insulin, as reported by the Wisconsin Epidemiologic Study of Diabetic Retinopathy (WESDR) [57].

## 4. Heterogeneity of DR Phenotypes

The classic view of DR suggests that this disease manifests slowly from no DR for a period of 8-10 years, to mild NPDR, to moderate NPDR, and culminates in PDR. However, DR has variable phenotypes with different progression rates. Not all diabetics develop vision threatening complications such as in the case of PDR or DME. Similarly, not all diabetics develop DR even in spite of the long duration of diabetes. What are the other factors which may influence the rate of progression or severity of the DR phenotypes? In this section, we discuss the variation of the natural course of the disease which cannot be solely be explained by the control of systemic factors, or duration of diabetes.

### 4.1. Are PDR and DME Two Distinct Diseases?

**Figure 4:**
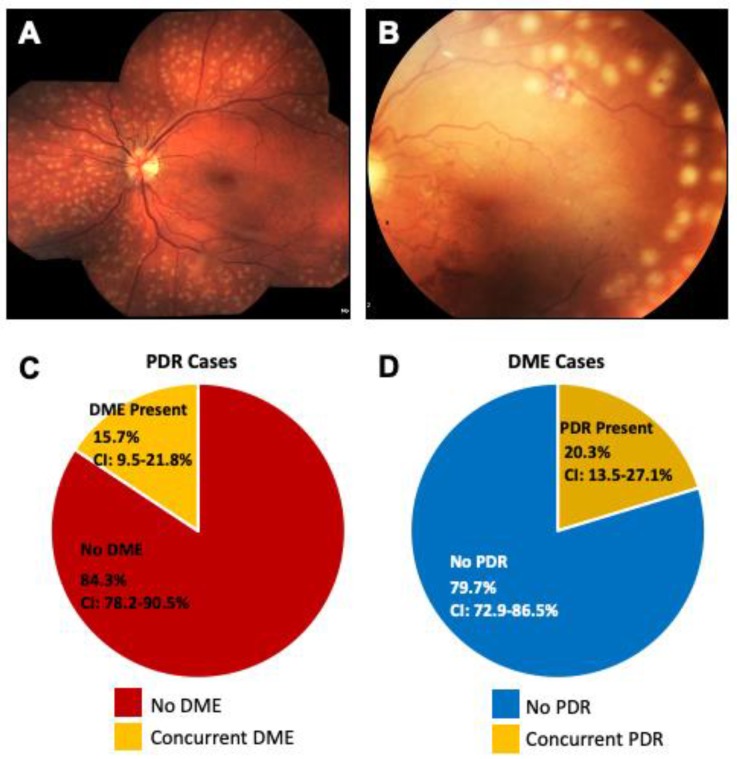
Coexistence of PDR and DME. (**A**) Fundus photograph of a PDR patient one day after vitrectomy with endolaser showing the macula without any exudates or edema, (**B**) Fundus photograph of another PDR patient with laser marks showing absence of any macular edema, (**C**) Pie chart showing co-existence of proliferative diabetic retinopathy (PDR) and diabetic macular edema (DME). In PDR patients, concurrent DME features were seen in only 15.7% of patients, the remaining 84.3% of PDR patients did not have any concurrent DME. (**D**) In DME patients, 79.7% of patients did not have any concurrent neovascularization. The remaining 20.3% of DME patients did not exhibit features of PDR. **CI**; Confidence Interval.

This question of “phenotypic heterogeneity” in DR remains a mystery. Vascular endothelial growth factor (VEGF), a potent proangiogenic and vasopermeability factor, plays a key role in neovascularization (PDR) and hyperpermeability (plasma leakage in DME) [53,54,58]. Yet, cases of PDR patients with extensive new vessels who have no macular edema in spite of high VEGF levels have been reported (Figure 4A,B). If VEGF causes increased leakage, why do not all PDR patients present with DME at the same time? Conversely, cases of DME patients with hard exudates and leakage, but no sign of neovascularization in spite of high levels of VEGF have also been reported. If VEGF causes neovascularization, why do not all DME patients develop neovascularization or PDR at the same time? This unique phenotypic heterogeneity in DR raises many questions.

PDR and DME have unique characteristics (neovascularization and retinal thickening, respectively) that clearly demarcate each phenotype. While DME and PDR can and do occur together, they mostly progress independently of each other, if they manifest at all. Our own retrospective cross-sectional study of 165 eyes with a new diagnosis of PDR (active neovascularization) shows that only 15.7% of eyes of patients with PDR had concurrent DME (95% CI 9.5%–21.8%) (Figure 4C) [59]. The confidence interval (CI) gives an estimated range of values which is likely to include an unknown population parameter. Similarly, only 20.3% of eyes of 166 eyes with new diagnosis of DME had concurrent retinal neovascularization, or PDR (95% CI 13.5%–27.1%) (Figure 4D). Stratified risk factor assessment demonstrated that neither gender, age, type of diabetes, HbA1C, mean arterial pressure nor LDL control were statistically significant in the development of DME in the PDR patients, or PDR in DME patients. While the genetic evidence that PDR and DME are indeed two distinct diseases has yet to emerge, our findings reveal that the clinical phenotype of DME can manifest independent of PDR development. At this point, it remains unclear why DME can appear in any stage of DR, but this phenomenon indicates that each disease phenotype has independent, perhaps genetic, risk factors. Indeed, studies already indicate that there may be genetic risk factor for DME. Recently, Gao et al. found that non-Hispanic blacks were three times as likely to develop DME compared to non-Hispanic whites [60].

### 4.2. Variable Drug Response to Treatment

The efficacy, as evaluated by many clinical trials, has made intravitreal anti-VEGF therapy a valuable treatment for DR in treating both PDR and center-involving DME [61,62,63]. However, despite the success of anti-VEGF therapy in restoring visual acuity in PDR, success has been limited in treatment for DME, with results from the DRCR Protocol I trial showing persistent macular thickening in 50% of patients [62]. A key feature of DME is the disruption of the blood-retinal barrier, whereas neovascularization is the hallmark of PDR. While VEGF has been shown to play a central role in both DME and PDR development, the variable treatment response of DME to anti-VEGF therapies indicate that these phenotypes may be two distinct diseases, each driven by distinct molecular mechanisms [64,65].

**Figure 5:**
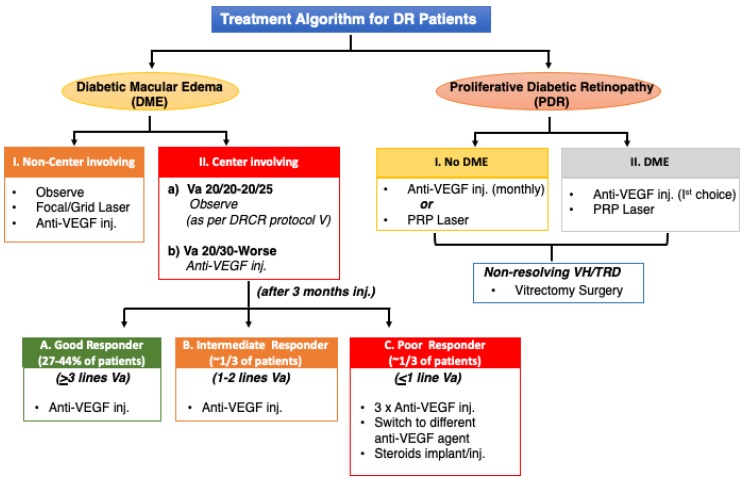
Treatment Algorithm for Diabetic Retinopathy Patients. Flow diagram outlining the various phenotypes of DR which are treated with anti-VEGF injection and laser. Treatment plan is carefully selected based on individual patient. **VEGF,** Vascular endothelial growth factor; **Va**, Visual acuity; **PRP**, Panretinal Photocoagulation **VH**, Vitreous hemorrhage; **TRD**, Tractional Retinal Detachment.

The response to anti-VEGF therapy is distinct in PDR as compared to DME (Figure 5). Nearly all PDR patients respond well with complete regression of new vessels with one or two anti-VEGF injections, whereas such a robust effect is rarely seen in DME patients [66]. The response of anti-VEGF drugs in DME is variable with only 30–40% patients responding well to treatment [56,62]. The variability in treatment responsiveness or differential efficacy of anti-VEGF drugs in PDR and DME suggests that there may be separate molecular pathways involved in the development of each phenotype.

All three major trials (DRCR, RIDE/RISE, VISTA) with anti-VEGF drugs have shown that only 33-40% of DME patients show 3-line visual acuity improvement [67,68,69]. A post hoc analysis of the DRCR Protocol I data reveals that 30–40% of patients with DME do not completely respond to anti-VEGF therapy [56,62]. Inter-individual variation in responsiveness (‘good’ vs. ‘poor’ responders) to anti-VEGF therapy in DME may be attributable in part to genetic variants. Interestingly, in a recent small study, the presence of the *VEGF* polymorphism C634G predicted a ‘good response’ outcome to anti-VEGF therapy [70]. However, these findings have yet to be confirmed in follow-up studies.

## 5. Clinical Evidence of Genetic Factors in DR Phenotypes

Multiethnic cohort studies have estimated a higher prevalence (two-fold) of diabetes and retinopathy in Natives (American Indians/Alaskan) and Hispanics as compared to non-Hispanic Whites [71,72,73]. These ethnic differences can be further seen within the same ethnic group where the Pima Indians in Arizona have the highest prevalence of diabetes along with Navajo and Sioux Natives [74,75,76]. These observations were independent of glycemic control and measured environmental factors. Additionally, In the Los Angeles Latino Eye Study (LALES), Native American ancestry in Latinos with type 2 diabetes was found to be significantly associated with severe NPDR or PDR [60]. The reason for disparities in rates and severity of DR in these ethnic groups remains unknown.

### Ethnic Differences in Disease Manifestation

In our own study of ethnic differences in DR, we studied a series of 1458 fundus photographs of American Indian and Alaska Native populations served by the Indian Health Service. Our analysis determined that the majority (90%) of DME cases in American Indian/Alaska Natives had mild, “focal” type, non-center-involving DME with few hard exudates and no central retinal thickening (Horton and Das, unpublished observations, 2017). The remaining 10% of patients had the “diffuse”, center involving DME, which is more prevalent among Hispanics. These differences in the type of DME (focal or diffuse) play an important role in treatment response. Focal DME treatment with focal/grid photocoagulation is needed in the majority of these patients and rarely requires anti-VEGF therapy. However, those with diffuse center-involving DME are typically treated with anti-VEGF injections.

## 6. Are Systemic Factors Always Related to Severity of DR?

In 1984, the Wisconsin Epidemiologic Study of Diabetic Retinopathy (WESDR, III) revealed that the severity of retinopathy was related to longer duration of diabetes, younger age at diagnosis, higher glycosylated hemoglobin levels, higher systolic blood pressure, use of insulin, presence of proteinuria, and small body mass [26,27]. Years later, in 1993, the Diabetes Control and Complications Trial (DCCT), showed that intensive glycemic control could prevent development as well as slow down the progression of microvascular complications, establishing glycemic control as a key risk factor for DR progression in type 1 diabetes [28]. Shortly after in 1996, the Early Treatment Diabetic Retinopathy Study (ETDRS, Report 22) demonstrated that elevated serum lipid levels were associated with the presence of retinal hard exudates in persons with retinopathy, and suggest that regulating lipid levels may decrease the risk of hard exudate formation and associated vision loss in patients with DR [77]. Then in 1998, the UK Prospective Diabetes Study (UKPDS) Group showed that tight control of blood pressure reduced the risk of DR progression and deterioration in visual acuity, confirming original findings of the link between hypertension and DR [78].

Together, these landmark epidemiological studies, and numerous confirmatory studies thereafter, helped solidify duration of diabetes, hyperglycemia, hypertension, and hyperlipidemia as the major risk factors associated with DR, establishing metabolic dysfunction as a key determinant of this disease. However, while these systemic factors correlate with slowed progression, emergent studies are consistently showing progression of DR independent of metabolic dysfunction. In fact, a closer statistical analysis at the original findings from the DCCT, by the DCCT group, reveal that the total glycemic exposure (HbA1C and duration of diabetes) accounts for only 11% of conferred variation in risk for retinopathy, and “other factors” may be responsible for the rest 89% of the risk variation among subjects independent of HbA1C levels [79]. These “other factors” have been attributed to genetic and environmental factors that may determine the overall risk of developing complications. Thus, the proceeding studies have been selected to invite readers to challenge the current understanding of the metabolic risk factors associated with DR, and point to other factors as determinants of DR.

### 6.1. Duration of Diabetes

The duration of diabetes is the most consistently cited risk factor associated with the development of DR. The WESDR study revealed that prevalence of any DR was ~97.5% in type 1 diabetics and 80% in type 2 diabetics in persons with diabetes with duration of diabetes of 15 years or more [26,27]. Despite large-scale studies from the WESDR, the Joslin 50 Year Medalist Study, in which type 1 diabetes patients who have survived more than 50 years of diabetes (Medalists) are followed, reports that about 40.2% of patients did not develop any retinopathy, or developed mild NPDR and only 48.5% of patients in this cohort advanced to PDR in their lifetime [31]. A high proportion of the Medalists also remained free from other diabetic complications such as nephropathy (86.9%), neuropathy (39.4%) or cardiovascular disease (51.5%). Further, there was no significant relationship between the longitudinal HbA1C levels or blood pressure levels and the complications. These studies not only challenge duration of diabetes as a strong predictor for retinopathy, but also indicate that “protective factors”, perhaps genetic or environmental, which may exist and could possibly contribute to the slowing or prevention of retinopathy. Interestingly, proteomic analysis of retinas and vitreous in this same cohort has already identified photoreceptor-secreted retinol binding protein 3 (RBP3) as a protective factor from advanced DR [80].

### 6.2. Hyperglycemia

Since the initial findings by DCCT, numerous studies reveal that the effect of intensive glucose control on the progression of retinopathy is not understood [29,30,31]. The Action to Control Cardiovascular Risk in Diabetes (ACCORD) Eye Study, in particular, was forced to discontinue their glycemia trial after 3.7 years because of higher mortality rates in the tight glucose control cohort (HbA1c < 6.0%), indicating that although tight control is beneficial in slowing progression of retinopathy, too rigid of a control may be detrimental and have severe cardiovascular consequences [81]. The inconsistency of the hyperglycemia as a determinant of progression to the vision threatening DR phenotype was reported in the LALES Study in which the HbA1C level was not found to confer any additional risk in progression to the PDR phenotype in a cohort of 1115 Latino diabetic patients [30]. In another prospective study of a group of 380 African Americans, the association between HbA1C level and PDR did not reach statistical significance [82]. Interestingly, both these studies found the duration of diabetes was consistently the strongest risk factor in progression to PDR. Similarly, several studies of DME patients have shown that there is no statistically significant difference in average HbA1c levels in patients with and without DME [83,84,85]. If higher HbA1c levels were strongly correlated with DME severity, one would be inclined to think higher HbA1c should lead to increased macular thickness. However, these studies refute this idea and further question the role of hyperglycemia in disease progression.

### 6.3. Hypertension

Blood pressure has been shown to be a strong risk factor for DR. The UK Prospective Diabetes Study Group (UKPDS) of type 2 diabetic patients showed that tight blood pressure control (achieved with use of angiotensin converting enzyme inhibitor or β blocker), compared with less tight control (no treatment), demonstrated reduced risk for progression of DR by 34% [78]. However, the benefit of controlling blood pressure has not been confirmed in other large studies [83,86,87]. In the ACCORD Eye Study, if patients were normotensive at the onset of DR, there was little benefit noted in reduction of retinopathy in decreasing systolic blood pressure (<120 mmHg) [81]. Additionally, the Action in Diabetes and Vascular Disease: Preterax and Diamicron Modified Release Controlled Evaluation (ADVANCE) study determined that there was no evidence of a beneficial effect of intensive blood pressure control on the progression of diabetic retinopathy [88]. In several studies, the blood pressure level was not significantly associated with the development of DME [83,86].

### 6.4. Hyperlipidemia

Lipid levels and their association with DR have been studied extensively. Observations from the ETDRS evaluating the relationship between serum lipid levels, retinal hard exudates, and visual acuity in patients with retinopathy demonstrated that patients with either elevated serum cholesterol or serum low-density lipoprotein cholesterol levels, were twice as likely to have retinal hard exudates as compared to patients with normal levels [77]. Additionally, the ETDRS reported that these patients were also at higher risk of developing hard exudates during the course of the study. These findings solidified elevated serum lipid levels as a risk factor for DR. However, more recent studies propose that there is no relation of lipid levels to DR [83,86,87,89]. A meta-analysis of 21 randomized controlled trials investigating the correlation between DME and dyslipidemia, could not affirm an association between lipid levels and DME [90]. This study additionally revealed that serum lipid control had no effect on DME progression as demonstrated by similar degree of severity in DME patients given lipid lowering treatment compared to those given placebos. 

In summary, all the preceding studies challenge the widely accepted risk factors and their association to DR. Conversely, they also point to the possibility of other factors, perhaps genetic, involved in the incidence, severity, and progression of the DR phenotypes. Furthermore, just as genetic factors may be involved in ‘protecting’ some diabetics from developing the disease, they may also dictate at what stage DME or PDR will appear, if at all.

## 7. Early Studies Point to the Role of Genetics in DR

The first indication of the role of genetics in DR came from early familial clustering studies, published well before the 1984 WESDR study implicating metabolic factors as key determinants of DR [91]. In this study of concordant (both diabetic) and discordant identical twins (only one diabetic) with at least 15 years duration of diabetes, findings indicated that genetics was a heritable trait for retinopathy. This was determined after observations of severe retinopathy with strikingly similar progression were seen among 12 of 13 concordant twin pairs compared to milder retinopathy in 5 of 10 discordant twins [91]. Additionally, the underlying role of genetics has been studies in Mexican American and non-Hispanic white populations, demonstrating higher prevalence of severe diabetic retinopathy among Mexican Americans compared to non-Hispanic whites, even after logistic regression control for duration of disease, hyperglycemia, age, and blood pressure [72,92]. Additional evidence of the underlying role of genetics in DR was further seen in the incidence of severity, where only 50% of type 1 diabetic patients progress to the more advanced proliferative stage of retinopathy (PDR) in their lifetime [27]. In the remaining 50% of these patients, the disease did not progress further in spite of the long duration of diabetes.

## 8. Leveraging Gene Mapping and Previous Genetic Studies

Advancements in technology have yielded an unprecedented boom in the study of the role of genetics in human disease [93]. Prior to single nucleotide polymorphism (SNP) genotyping, genetic studies of DR were rooted in linkage analysis and candidate gene associations [74,94,95,96,97,98]. Today, microarray/biochips are used to identify single nucleotide changes in a DNA sequence (SNPs), and attributing them to increased susceptibility for disease [99,100]. Here, we have chosen to highlight the findings of the largest, to our knowledge, DR genome-wide association study (GWAS) to date. In this recent study, eight European (*n* = 3,246) and seven African American cohorts (*n* = 2,611) including European, Asian, and Hispanic subjects were meta-analyzed, with and without liability threshold modeling for glycemic control and duration of diabetes [101]. Using the threshold for genome-wide significance (*p* < 5 × 10^-8^), several SNPs where identified among PDR and no DR cohorts (rs115523882, rs139205645, rs17791488, rs184340784, rs142293996, rs17706958, rs80117617). The SNP rs115523882, seen in the African American PDR group, was found to be the most significant of the cohorts studied (*p* = 5.37 × 10^-9^). The authors highlight its location near the *GOLIM4* gene, which is implicated in changes in the Nlx3 binding motif, a known blood transcription factor and thereby infer its relevance to DR. However, these findings could not be reproduced in replication cohorts. Additional variants identified were further examined for enriched protein networks among the loci with highest statistical evidence for association with DR, finding only one variant in the European discovery cohorts. However, the intronic variant in the nuclear VCP-like (*NVL*) gene identified also failed to reach genome-wide significance after meta-analysis in the replication cohorts.

In the last decade, genetic mapping has been used to identify over 75 DR-associated gene variants [102,103,104]. However, replicative studies in independent cohorts and confirmatory studies demonstrating the effect of these variants are still pending. Of the variants identified thus far, most have resulted in weak associations often attributed to the variability in case definitions of the various phenotypes analyzed, not distinguishing phenotypes within and between studies (NPDR vs. PDR, and/or DME), inconsistently defined controls, cohort heterogeneity (e.g., discovery and replication samples coming from different ethnic populations), and sample size. An additional limitation of these early studies is in the duration of diabetes of the cohorts studied, often limited to ~10 years in most. Based on studies by the WESDR group, nearly 100% of with type 1 diabetes and approximately 60% of patients with type 2 diabetes will develop retinopathy within the first 20 years of diabetes onset, indicating that disease development or progression after 20 years of diabetes is highly unlikely [105]. Thus, studies should be limited to include only patients who had a minimum of 20 years diabetes as controls, ensuring that a ‘null’ retinopathy diagnosis at the time studies are conducted is not a false negative. This limitation is often acknowledged and attributed to lack of availability of patients who meet these criteria.

Importantly, these studies provide a foundation for future approaches in the study of DR genetics. By addressing these limitations, genetic mapping has the potential to unveil previously unsuspected mechanisms underlying this disease which could be leveraged as novel therapeutic targets. The following questions and concepts represent a series of observations that are of interest to us. These questions, yet to be addressed in the field, illustrate how the role of genetics can be used to strengthen our understanding of DR pathogenesis.

## 9. Nonsequential Progression of DR

**Figure 6:**
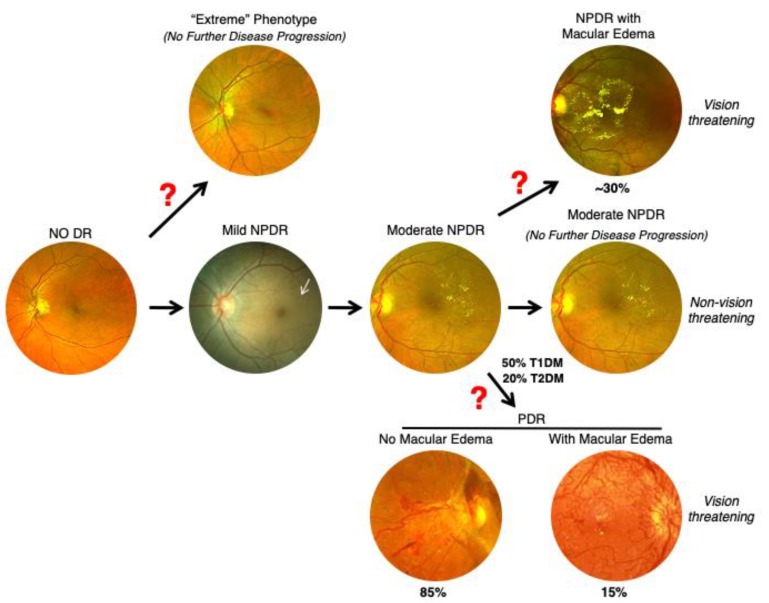
Proposed Course of Progression of Diabetic Retinopathy. Based on our clinical observations and large epidemiological studies, we propose that not every DR patient progresses through disease in the same sequence of events. After a period of no diabetes for 10 years, mild NPDR develops and over time advances to moderate NPDR. In some cases, there is no further disease progression. However, 30% of cases develop concurrent DME. About 50% of type 1 diabetics and 20% of type 2 diabetics develop PDR. In PDR patients, 15% develop concurrent macular edema while 85% never develop macular edema. Additionally, about 1–5% diabetic patients never develop retinopathy despite >20 years of diabetes (“Extreme” phenotype). Red question marks indicate divergence points which may be influenced by genetic factors.

The classic view of DR suggests that this disease manifests at different rates and in discrete stages of increasing severity (no DR, NPDR, to PDR) [37,38]. Based on our clinical observations and large epidemiological studies, we can infer that not every DR patient goes through the same sequence of progression to different DR phenotypes (Figure 6). After a period of no DR for about 8–10 years, one develops mild NPDR followed by moderate NPDR, out of which only 50% of type 1 diabetics and 20% of type 2 diabetics develop PDR [26]. It is unclear as to why not all NPDR patients develop PDR, despite longer duration of diabetes. Once PDR develops, ~15% of PDR patients may develop concurrent DME, while the remaining 85% of PDR patients never develop any macular edema [59]. Similarly, not all moderate NPDR patients develop DME. As the hemoglobin A1C level is not always significantly associated with development of PDR or DME, it is possible that further progression from mild-moderate NPDR to predominantly exudative changes (DME) or predominantly neovascular changes (PDR) are regulated by genetic determinants. Based on our results and other published work, we hypothesize, as previously noted, that a small subset of diabetic patients are protected, and further progression, if occurring, is slow and only advances to mild NPDR in spite of 20 years or longer duration of diabetes (Das, unpublished observations, 2017) [31]. These protected individuals are known as “extreme phenotype” cases of no DR.

## 10. Diabetic Nephropathy and Retinopathy

Many similarities between kidney disease, including diabetic nephropathy (DN) and chronic kidney disease, and DR exist. Often occurring in conjunction, DN and DR are both microvascular complications of diabetes, multifactorial diseases, and both have heterogenous phenotypes. Additionally, both are classically thought to progress in sequential stages of increased severity, and sometimes exhibit ‘rare’ cases of non-sequential disease progression [106].

**Figure 7:**
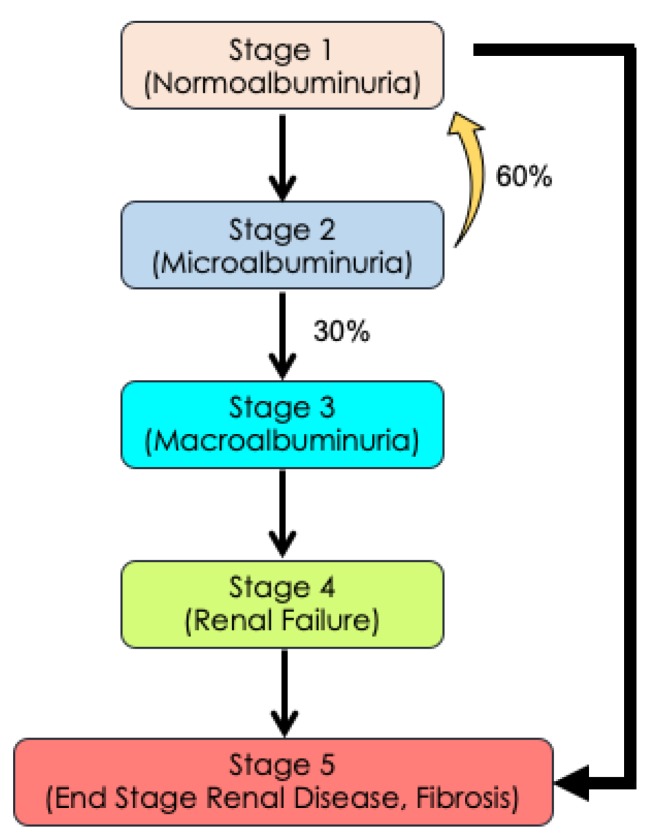
Diabetic Nephropathy has a Progressive and Continuous Heterogenous Phenotype. As Stage 1 (normoalbuminuria) of nephropathy progresses to Stage 2 (microalbuminuria), 60% of Stage 2 patients may revert to Stage 1 while 30% of Stage 2 patients progress to Stage 3 macroalbuminuria. The disease further progresses to Stage 4 (renal failure), culminating in Stage 5 (end Stage renal disease with fibrosis). A small number of Stage 1 patients may advance directly to Stage 5 without any signs of albuminuria. This progression may be genetically determined. In diabetic retinopathy, a similar phenomenon may occur where genetic determinates may influence whether patients will advance to PDR and/or DME.

In DN, the disease typically progresses through five discrete stages with clearly demarcated phenotypes ranging from renal hyperfunction with normoalbuminuria (stage 1), to microalbuminuria (stage 2), to macroalbuminuria (stage 3), to renal failure (stage 4), culminating in end-stage renal failure with fibrosis (stage 5) (Figure 7) [107]. As stage 1 of DN progresses to stage 2 microalbuminuria, 60% patients revert to stage 1. Further, only 30% of stage 2 patients progress to the next severe stage 3 macroalbuminuria, which eventually progresses to stages 4 and 5. However, reports show that some patients can directly progress from stage 1 directly to stage 5 without going through the albuminuria stage. This rapid advancement in disease progression was shown to be genetically determined [106]. In DR, a similar phenomenon can be observed where a patient develops mild NPDR, progresses to moderate NPDR, and advances to PDR without ever going through the DME stage. Moreover, the features of DME can occur at any stage of the disease, independent of PDR, and point to the possibility of DME and PDR being two distinct diseases.

## 11. Diabetic Retinopathy Genomics (DRGen) Study

Recognizing the value of gene mapping as a tool to understand the underlying architecture of disease, we have recently initiated the Diabetic Retinopathy Genomics (DRGen) Study, a collaborative effort between University of New Mexico (UNM) School of Medicine and Harvard’s Joslin Diabetes Center [108,109]. Using a well-defined, clinically supported phenotypic strategy, we seek to better understand the role of rare variants in DR progression, protection, and variable response outcome of anti-VEGF treatment in DME. Using whole exome sequencing (WES) technology, the coding region of all genes will be screened for associations with the clinical phenotypes of DR. Our interest lies in genes known to be involved in inflammatory and angiogenesis pathways, both of which are known to play a role in DR pathogenesis but have previously shown weak associations. A key feature of the DRGen study is our stringent classification of phenotypes used for the comparison among the groups of interest (1) no DR despite 25 or more years of diabetes, (2) severe PDR without concurrent or prior DME history, and (3) DME without concurrent or prior history of PDR.

Using the aforementioned study design, two cohorts were selected from the DRGen study population established at the UNM School of Medicine. Briefly, as a proof of principle, we analyzed an ‘extreme’ phenotype (no DR despite > 25 years of diabetes; n = 6) and an ‘advanced’ PDR phenotype (PDR case with history of vitreous hemorrhage within 15 years of diabetes; n = 6). All subjects were matched for gender and age. After obtaining informed consent, DNA was isolated from white blood cells. WES was conducted using the SureSelect All Human XT v5 exome kit, analyzed on the Illumina NovaSeq platform, followed by in-house downstream analysis pipeline to align the sequence reads. The variant list was custom filtered with minor allele frequency (MAF) < 5.0, and VCFtools (v0.1.13) was used to calculate fixation index (FST) statistics. FST analyses captured the population genetic distance between these two phenotypes and identified 114 genes, which showed at least one variant that had FST values of greater than 0.8 (FST > 0.8) for the two phenotype comparison which demonstrates that these genes may play an important role in the pathogenesis of DR [110,111].

**Figure 8:**
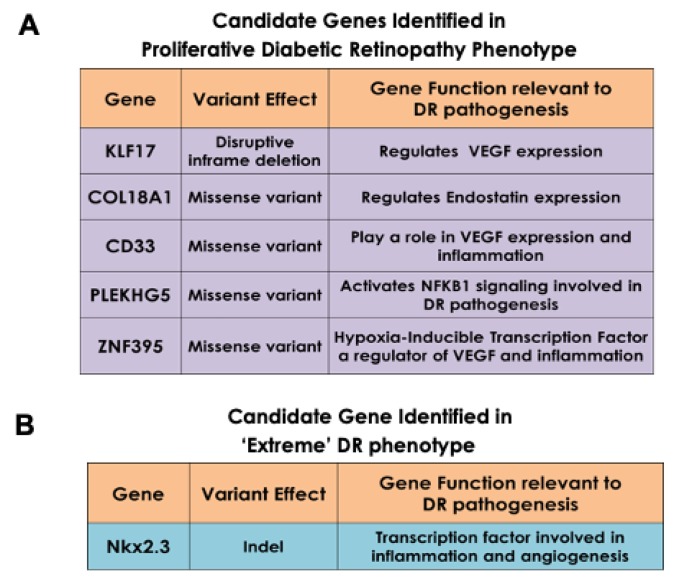
Newly Identified Genetic Variants Associated with Specific Phenotypes of Diabetic Retinopathy. (**A**) Enrichment of the ‘risk’ allele in five candidate genes in cases of advanced PDR patients revealed a disruptive in-frame deletion and four heterozygous missense variants. (**B**) A variant in the NKX2.3 gene was shared in the “extreme” DR phenotype (No DR) group.

**Figure 9:**
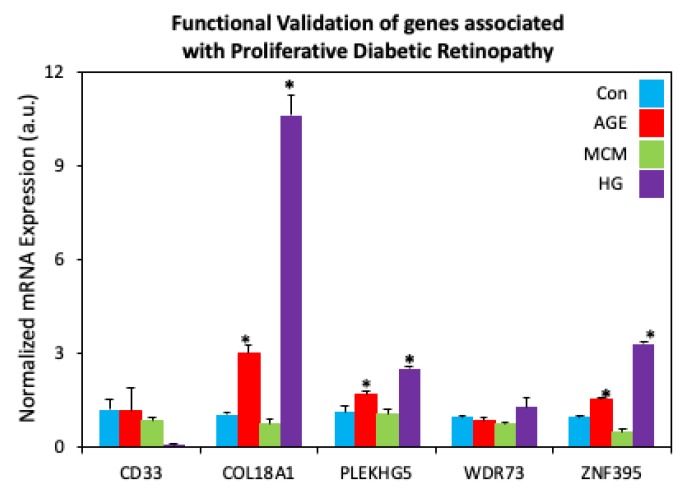
Functional Validation of genes associated with Proliferative Diabetic Retinopathy. Human retinal endothelial cells treated with advanced glycation end product-BSA (AGE, 500 μg, 3 days; Sigma Aldrich, St. Louis, MO), macrophage conditioned medium (MCM, 1:1 ratio, 24 h), or high glucose medium (HG, 30 mM, 7 days) revealed increased mRNA expression of COL18A1, ZNF395, and PLEKHG5 in AGE and HG-treated cells. * *p* < 0.05. Bars indicate average ± standard deviation.

**Figure 10:**
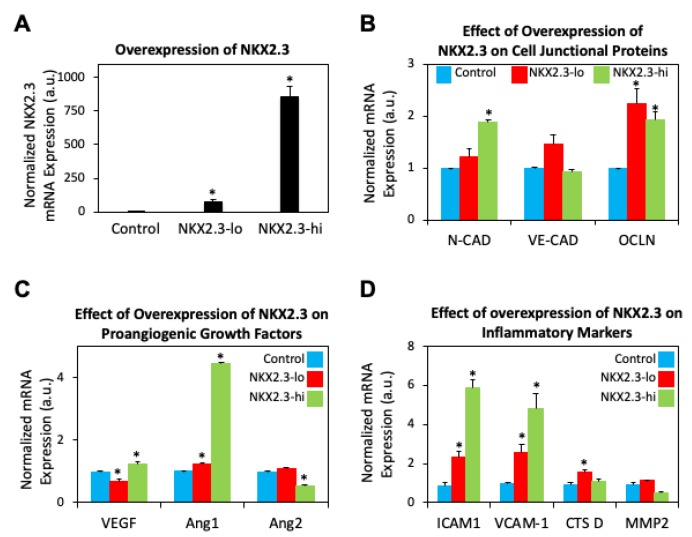
NKX2.3 gene associated with ‘extreme’ phenotype leads to mRNA expression changes in angiogenic and inflammatory markers. (**A**) mRNA expression of NKX2.3 was increased in human retinal endothelial cells (HREC) using varying concentrations of PCDNA plasmid (lo: 5 μg + 3.75 μL, hi: 5 μg + 7.5 μL; 24 h). (**B**) Overexpression of NKX2.3 in HRECs revealed increased mRNA expression of cell-cell junctional proteins N-cadherin (N-cad) and occludin (OCLN). (**C**) Changes in pro-angiogenic vascular endothelial growth factor (VEGF), angiopoietin 1 (Ang1) and angiopoietin 2 (Ang2) mRNA expression were also observed. (**D**) Overexpression of NKX2.3 in HRECs also revealed increased expression of inflammatory markers intracellular adhesion molecule-1 (ICAM-1), vascular cell adhesion molecule-1 (VCAM-1), and cathepsin D (CTS D). * *p* < 0.05. Bars indicate average ± standard deviation.

The enrichment of “risk” alleles in cases with MAF < 0.05% was tested, identifying four heterozygous missense variants and a frame shift mutation in the PDR group. Our preliminary findings revealed novel genetic variants Kruppel Like Factor 17 (*KLF17*), Zinc Finger Protein 395 (*ZNF395*), Myeloid cell surface antigen (*CD33*), Pleckstrin Homology Domain-Containing Family G Member 5 (*PLEKHG5*), and Collagen Type XVIII Alpha 1 Chain (*COL18A1*) in the ‘advanced’ PDR cohort (Figure 8A). These genes have been previously shown to be involved in the angiogenesis and inflammatory pathways, both implicated in DR progression [112,113,114,115,116]. Our analysis of the rare coding variants in our ‘extreme’ cohort (no DR) revealed variance in the NK2 Homeobox 3 (*NKX2.3*) gene (Figure 8B). NKX2.3 is a member of the NKX transcription factor family that has been shown to regulate genes involved in immune and inflammatory response, cell proliferation and angiogenesis [117]. These variants have not been studied well in the context of DR. However, our preliminary analysis of mRNA isolated from human retinal endothelial cells treated with high glucose, show increased expression of COL18A, ZNF395, and PLEKHG5 (*p* < 0.0001), providing a clue of the effect of hyperglycemia and inflammatory cytokines present in DR (Figure 9). Additionally, our overexpression studies of NKX2.3 show that NKX2.3 plays a role in regulating cell-cell junctional proteins, proangiogenic growth factors, and inflammatory markers involved in DR pathogenesis (Figure 10). Further validation of these variants is necessary to confirm our findings in larger number of patients with different DR phenotypes. Currently, the DRGen study is actively enrolling patients with the defined phenotypes (“extreme” phenotype with no DR, DME and PDR).

## 12. Future Perspectives

The last ten years have represented a period of intensified molecular genetic research in the field of DR. Warranted by the precedence, complexity, and potentially debilitating vision loss, many studies have attempted to understand the underlying role of genetics in this disease. While clinical evidence indicates that genetic factors are implicated in retinopathy, their precise role remains elusive. Nevertheless, continuous efforts remain focused on the identification of DR-associated novel genetic variants. For some complex human traits, we are now reaching the point where we can make accurate predictions. For example, based on the genomic sequence alone, complex traits such as height can be accurately predicted within a centimeter [118]. Similarly, the risk for few diseases such as Crohn’s disease and Parkinson’s disease may also be predicted. We are hopeful that similar level of accuracy may one day be available to assess the risk of DR, the type of DR, the severity, and the response to available therapies. These possibilities represent the “tip of the iceberg” for future care. However, arriving at a place where precision medicine is commonplace will require evolving our approach in the way we understand this disease.
